# Sex-Based Differences in Fatigue During Repeated Sprinting in 9- to 14-Year-Old Children Are Task- and Metric-Dependent

**DOI:** 10.3390/sports14030104

**Published:** 2026-03-05

**Authors:** Bilgin Ataş, İbrahim Can, Bouwien C. M. Smits-Engelsman

**Affiliations:** 1 Secondary School of Tuzluca Atatürk, Tuzluca, Iğdır 76900, Türkiye; tkdbilgin@gmail.com; 2 Department of Coaching Training, Faculty of Sports Sciences, Iğdır University, Iğdır 76000, Türkiye; ibrahim.can@igdir.edu.tr; 3 Physical Activity, Sport and Recreation (PhASRec, Focus Area), Faculty Health Sciences, North West University, Potchefstroom 2531, South Africa; 4 Department of Natural Sciences in Kinanthropology, Faculty of Physical Culture, Palacký University Olomouc, 2531 Olomouc, Czech Republic

**Keywords:** repeated-sprint ability, children, (pre)adolescence, fatigue, sex, metabolic, anaerobic capacity

## Abstract

Background: This study investigated sex-based differences in sprint performance and fatigue among 9–14-year-olds using two repeated-sprint protocols: the Muscle Power Sprint Test (MPST; 6 × 15 m) and the Children’s Repetitive Intermittent Sprint Performance test (CRISP; 6 × 30 m). Additionally, four fatigue metrics were compared: decay (first vs. last sprint), fatigue index (fastest vs. slowest sprint), sprint decrement (ideal vs. actual total time), and slope IP (regression slope across all sprints). Methods: A total of 140 children (9–14 years; 70 females, 70 males) performed the two tests in randomized order. They completed the six sprints per test with 10 s of recovery between each sprint of either 15 or 30 m. Fatigue metrics were calculated for each test based on the sprint times. Results: Running speed was higher in CRISP than in MPST, and males outperformed females in both tests. In the MPST, fatigue metrics did not differ significantly by sex. In contrast, all fatigue indices in CRISP were significantly greater in females, indicating higher fatigue despite slower sprinting compared to males. Among the fatigue metrics, slope IP correlated most strongly with decay, while associations with fatigue index and sprint decrement were weaker. Conclusions: Fatigue assessment is more sensitive over longer sprint distances. Females demonstrated significant fatigue in CRISP, indicating that fatigue is task-dependent. The slope metric, which incorporates all sprints, offers a robust fatigue measure for group comparison, while decay remains a practical alternative for field settings.

## 1. Introduction

Early adolescence, typically occurring between the ages of 10 and 14 years, is a critical developmental phase during which motor skills are refined, physical fitness foundations are established, and lifelong health behaviors are shaped [1]. Physical activity during this period supports not only physical fitness but also cognitive, metabolic, and psychosocial well-being [2,3]. Recently, interest has grown in school-based physical activity programs that incorporate high-intensity, short-duration exercise formats tailored to children’s developmental characteristics. Among these, repetitive sprint training, characterized by short bursts of maximal effort interspersed with brief recovery, has been shown to improve both cardiometabolic markers and cognitive functions such as working memory [4]. These exercises also align with children’s natural play and sports patterns, which are typically impulsive, intense, and brief, contributing to high levels of engagement and motivation. Repeated-sprint tests are expected to reflect the physiological stress in these kinds of activities [5,6]. As such, repeated-sprint ability (RSA) is increasingly recognized as a key component of youth physical fitness, relevant not only for athletic development but also for promoting healthy lifestyles.

In children, performance decrement across repeated sprints is generally lower than in adults, despite their lower peak-power outputs [7]. This resilience is linked to developmental differences in energy system contributions: children produce less lactate and rely more on aerobic metabolism, which limits the metabolite accumulation typically associated with fatigue in adults [8]. Additionally, faster recovery of the ATP-PCr system in children supports repeated short-duration efforts, even when anaerobic capacity is limited [9].

Repeated-sprint ability (RSA) is particularly relevant for assessing physical fitness in youth, as children are naturally exposed to short, high-intensity bursts of activity, such as sprinting, jumping, and throwing, during play and sport [10]. These efforts contribute to the development of muscular strength, coordination, and anaerobic endurance, supporting both healthy growth and long-term performance potential [11]. Prepubescent children generally exhibit lower anaerobic capacity and depend more heavily on aerobic metabolic pathways during submaximal exercise compared with postpubescent children [12]. Their metabolic response to exercise is characterized by lower lactate accumulation following high-intensity exercise [13] as well as higher rate of fat oxidation relative to postpubescent children and young adults [14,15]. Children regain performance quickly between sprints, even with short rest periods. Additionally, less absolute and relative peak power and mean power during repeated sprints were observed in prepubescent children when compared to adult men [16].

Multiple factors, including sprint duration, number of repetitions, and recovery intervals, shape the energy demands during repeated-sprint ability. As sprint repetitions accumulate, anaerobic energy contribution declines, but performance does not decrease proportionally, an effect attributed to increased aerobic support [17]. Oxygen consumption and lactate accumulation rise especially when recovery is short [18], indicating that RSA involves both anaerobic and aerobic mechanisms. Beyond energy metabolism, fatigue in RSA also reflects central and peripheral neuromuscular processes, influenced by factors such as motivation, pacing, and perception of effort [19].

Short sprint distances of 15 m and 30 m are developmentally appropriate for children because they reflect the way they naturally perform high-intensity movements and capture two distinct phases of youth sprinting. Children’s spontaneous physical activity consists of brief, intermittent bursts, with most intense actions lasting only a few seconds and nearly all under 15 s; thus, 15 m sprint represents the acceleration phase that dominates their habitual movement patterns. In contrast, children typically reach near-maximal running velocity only after approximately 25–30 m, making a 30 m sprint a functionally distinct measure of maximal-speed or speed-maintenance ability. Together, these distances align with age-specific neuromuscular development, reflect naturally occurring activity patterns, and allow meaningful differentiation between acceleration capacity and emerging maximal-velocity performance in children aged 9–14 years [20,21,22,23].

Despite widespread use of RSA protocols in pediatric populations, little is known about how sprint distance influences fatigue-related performance decay, especially when comparing sexes. Understanding whether sex differences in RSA fatigue are task-dependent is essential, as females may demonstrate distinct fatigue patterns despite having lower absolute power output.

In repeated-sprint protocols, performance typically declines across successive efforts. This decline, commonly referred to as “fatigue”, is often quantified using measures such as the fatigue index, sprint decrement, or performance slope. While the term “fatigue” is widely used in this context, it represents a multi-factorial phenomenon involving physiological, neuromuscular, and motivational elements [24]. For clarity and consistency with the exercise science literature, in this study, we define fatigue as the observable decline in sprint performance across repeated efforts, primarily attributed to incomplete recovery between sprints.

Accurately quantifying fatigue during repeated-sprint ability is essential for understanding the physiological demands of high-intensity efforts in youth. However, the literature presents a range of fatigue metrics, each capturing slightly different aspects of performance decay [25]. These metrics often yield divergent results, complicating the interpretation and comparison of findings across studies [26,27]. Some indices emphasize the difference between the best and worst performance (e.g., fatigue index), while others consider the full performance trajectory (e.g., slope), which may better reflect how fatigue accumulates across repetitions [28,29]. Some authors even question if fatigue index is a worthwhile measure of repeated-sprint ability based on its large variability [28].

This methodological variability has practical implications, particularly when assessing training adaptations or developmental differences. Moreover, the extent to which these different metrics capture sex-based differences in fatigue remains unclear. While males often show higher sprint performance due to sex-related increases in muscle mass and power during adolescence [11], it is less well understood whether fatigue, as measured by different indices, manifests differently in males and females. Previous studies in younger children suggest developmental differences in anaerobic metabolism and recovery [8,30,31] between age groups, highlighting the need to assess older children separately. This study, therefore, compares multiple fatigue metrics across two protocols in males and females to clarify how task demands influence the interpretation of RSA performance and fatigue in this age group. This study aims to investigate how fatigue develops during repeated 15 and 30 m sprints in 9- to 14-year-old males and females. By comparing multiple fatigue indices across both protocols, we examine whether sex-based differences in RSA are task-dependent and explore which fatigue metrics are most sensitive and informative in this population.

## 2. Methods

### 2.1. Participants

All children were in grades 4–8 (between the ages of 9 and 14 years) at the selected two schools and we received signed written informed consent from their parents; no children with history of a condition that could negatively affect running performance were eligible to be included in this study (n = 140). The study received ethical approval from the Scientific Research and Publication Ethics Committee of the University of Iğdır (SRPEC approval no 33/12, September 2024) and was performed in accordance with the Declaration of Helsinki. Permission was obtained from the principal of the school and designated education authorities in the Iğdır Province of Turkey. The study sample size was calculated based on an expected medium effect size (d =  0.60) difference between males and females. Power analysis indicated that a total sample size of 126 was needed for a power of 90%, while alpha was set at 0.05. The G-power analysis software version 3.1 was used for the sample size calculation [32].

### 2.2. Demographic and Anthropometric Characteristics

Demographic data including age and sex were collected. Height was measured using a steel tape measure (Fisko Tri-Lok, Essex, UK) in the anatomical position and without shoes, while body weight was measured using an electronic scale (Medishop BY-810, Montevideo, Uruguay) without shoes and in light clothing. Body mass index (BMI) was calculated using the formula based on body weight and height (kg/m^2^).

### 2.3. Muscle Power Sprint Test (MPST)

During the MPST, each participant performed six runs at top speed over a 15 m course [33]. The rest time between consecutive runs was 10 s. The time taken to complete each run was measured using a stopwatch. Test–retest reliability using a manual stopwatch has been reported in two different studies to be good with ICC values of 0.98 and 0.90. Also, intertester reliability was excellent at 0.97 [34,35].

### 2.4. Children’s Repetitive and Intermittent Sprinting Performance (CRISP)

The CRISP test [30] consists of six sprint runs performed at maximal speed over 30 m with short recovery periods (10 s) between each run.

### 2.5. Procedures

Participants were tested in small groups during their physical education classes. Two researchers, trained in the correct administration of the protocol, conducted the tests. The procedures were explained verbally and demonstrated at the start of each session. Each participant received a demonstration before beginning each task. Participants completed a general warm-up protocol consisting of 10 min of low-intensity jogging and 5 min of dynamic stretching exercises targeting both the upper and lower body. After this, the standardized one-minute sprint warm-up was applied before each test using short sprints. Furthermore, the participants were allowed one practice trial to familiarize themselves with the tasks.

Participants were instructed to run as fast as possible from one line to the other, without slowing down before crossing the finish line. Sprint times were recorded manually using an electronic stopwatch (TFA Dostmann, Ettenheim, Germany), and verbal encouragement was provided throughout the testing. The order in which the two sprint protocols were administered was randomized.

Participants attended two repeated-sprint test sessions, each separated by a 48 h recovery interval. This interval was implemented to ensure adequate physiological recovery between tests. All testing measurements were scheduled at the same time of day for each participant to reduce potential circadian rhythm effects on performance outcomes and were performed outside on a non-slippery clay playground in a public school.

### 2.6. Fatigue Calculations

The following indices of performance were calculated:

1. Mean running time, calculated as the mean of time needed for the six sprints.
2. Mean running speed, calculated as the mean of the running speed over six sprints (speed = distance ÷ time).
3. The simplest metric for fatigue is comparing the time needed for the first and last run, decay start to finish (decay), using the following equation: Decay (%) = ((First Run − Last Run)/(First Run)) × 100.
4. The second option is comparing the fastest and slowest run, which uses the extremes in performance called the fatigue index (FI), which was calculated from the following equation: FI (%) = ((Fastest run-Slowest run)/(Fastest run)) × 100.
5. The third metric compares the option that the best run was accomplished every time (ideal sprint performance) relative to the actual time needed for the six runs, the sprint decrement (DECR), which was calculated from the following equation: DECR (%) = (Total Time/(6×Fastest Run)) × 100.
6. Lastly, the slope of the individual performance time decrement (slope IP) over the six runs was calculated using linear regression. For each subject, a linear curve was fitted to the data points of the running time over the six runs, and a least squares calculation was used to determine the goodness of fit (R2). The slope and fit calculated from each of these regression lines were examined using repeated measures ANOVAs. These regression estimates provided a detailed description of the temporal pattern of performance per participant. The slope reflects the extent to which performance becomes slower when the number of runs increases (fatigue) and if this slope, in that participant, is significantly different from zero. Because it is the only metric that accounts for the individual performance across all 6 runs, it is seen as the better fatigue metric.

For ease of interpretation, the fatigue indices were made positive so that higher values for decay, FI and DECR indicate more fatigue.

### 2.7. Data Analysis

Statistical analyses were performed with SPSS 30.0 (SPSS Inc., IBM Company, Armonk, NY, USA). Data were checked for normality. Extreme values (>3 SD), determined over the whole sample, were taken out: for sprint decrement MPST, this resulted in excluding data points of 1 subject, for decay CRISP data points of 1 subject, for slope MPST and for slope CRISP data points of 2 subjects were excluded. Fatigue index (FI) and sprint decrement (DECR) were not normally distributed. To verify whether the fatigue indices were larger for CRISP than for MPST, Wilcoxon signed-rank tests were performed. To examine if females responded differently to the sprint protocols than males, Mann–Whitney U tests were performed. Effect sizes (Cohen’s d) based on Mann–Whitney U tests were calculated using the formula by Fritz et al. [36]. The magnitude of effect size was considered as trivial d < 0.2; small 0.2 < |d| < 0.5; moderate 0.5 < |d| < 0.8; or large |d| ≥ 0.8 [37]. Lastly, the strength of relationship between fatigue variables was analyzed using Spearman rank-order correlations. Statistical significance was set at *p* < 0.05.

## 3. Results

### 3.1. Participants Characteristics

In total, 140 students (half males, half females) participated in this study. Age by sex distribution is given in Table 1. The mean age for the males was 11.5 (SD ± 1.6, range 9–14) and 11.1 years (SD ± 1.5, range 9–14) for the females (t 1.31, *p* = 0.19). Moreover, no significant sex differences were found in weight (t −0.09, *p* = 0.93; males 38.4 (10.2), females 38.5 (9.6) kg), height (t 1.67, *p* = 0.10; males 1.47 (0.11), females 1.44 (0.9) m) or BMI (t − 1.16, *p* = 0.25; males 17.6 (3.9), females 18.4 (4.0)).

### 3.2. Repeated-Sprinting Ability (RSA)

Means, medians, SDs and IQRs of all RSA outcomes per sex are given in Table 2. Statistics for the test (MPST vs. CRISP) and sex comparison are given in Table 3.

### 3.3. Running Time and Speed

**Figure 1:**
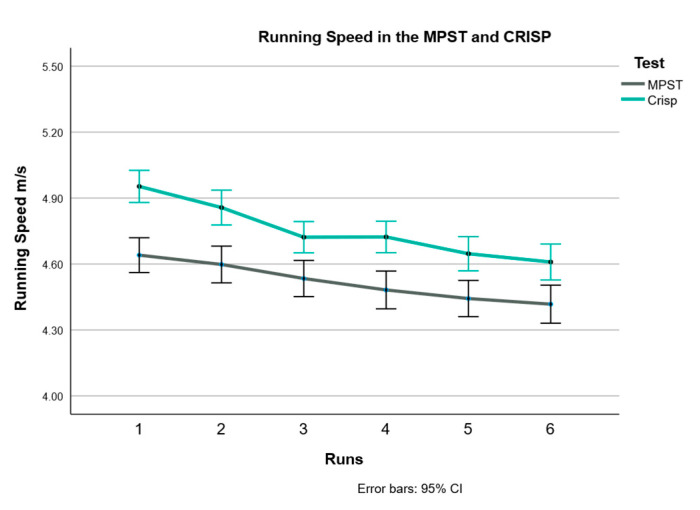
Running speed (m/s) in the two tests over the six runs. Higher speed was seen in the CRISP compared to the MPST (*p* < 0.001).

**Figure 2:**
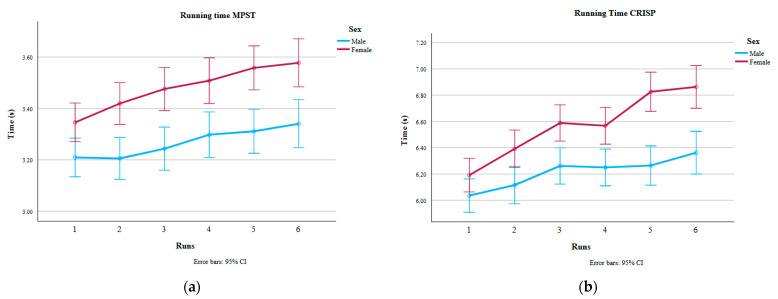
(**a**) Figure on the left shows running time (s) in the MPST over the six runs for males and females. Longer times were observed for the females (*p* < 0.001). (**b**) Figure on the right shows running time (s) in the CRISP over the six runs for males and females. Longer times were observed for the females (*p* < 0.001).

As expected, running time increased with distance: it took participants longer to complete the 30 m CRISP (mean 6.39 s) than the 15 m MPST (mean 3.37 s; *p* < 0.001). Despite the longer distance, participants ran at a higher average speed during the CRISP (4.74 m/s;) compared to the MPST (4.48 m/s; *p* < 0.001), as shown in Figure 1. In both protocols, females ran significantly slower than males, as illustrated in Figure 2a (MPST) and Figure 2b (CRISP). Detailed statistical outcomes are presented in Table 3.

### 3.4. Decay Start Finish (Decay)

When fatigue was calculated as the percentage decrease in speed from the first to the last sprint, significant differences were found between the two protocols. Participants showed a decline of 8.5% in the CRISP test compared to 5.7% on the MPST (*p* = 0.001). Sex differences in the MPST did not reach statistical significance (*p* = 0.06; d 0.322); however, females demonstrated a significantly greater decline than males in the CRISP test (*p* < 0.001; d 0.599).

### 3.5. Fatigue Index (FI)

Fatigue was also assessed by calculating the percentage difference in speed between each participant’s fastest and slowest sprint. The results showed no significant difference between the two protocols (MPST: 14.1%, CRISP: 14.2%; *p* = 0.39). However, in the CRISP test, females exhibited a significantly greater decline in speed compared to males (*p* = 0.004; d 0.50), suggesting higher fatigue. No such sex difference was observed in the MPST (*p* = 0.19).

### 3.6. Sprint Decrement (DECR)

To determine the sprint decrement, we calculated the difference between the participants’ actual total sprint time and the hypothetical ideal time, assuming they could repeat their best sprint six times. No significant difference was found between the CRISP and MPST protocols in terms of overall sprint decrement (*p* = 0.63). However, in the CRISP test, the females showed a greater deviation from their ideal sprint time (8.1%) compared to males (5.7%), indicating a statistically significant difference (*p* = 0.004, d 0.504). In contrast, during the MPST, the decrements were 6.9% for females and 6.2% for males, which was not statistically significant (*p* = 0.15).

### 3.7. Slope of Individual Performance Decrement (Slope IP)

The regression analysis of individual performance across the six sprints produced several key indicators. The y-intercept aligns with the estimate of the running time in the participant’s first run. The slope reflects the rate of performance decline across repeated sprints. The R^2^ value indicates how well the linear model fits the participant’s sprint data and serves as an indirect indicator of performance consistency. Additionally, it was determined for each participant whether their slope significantly differed from zero. No significant sex difference in slope was observed during the MPST (*p* = 0.28), whereas a clear sex difference emerged in the CRISP protocol (*p* < 0.001; d 0.744), with females showing a steeper slope than males. This suggests that females experienced a pronounced decline in running speed across the CRISP sprints. When comparing the two protocols, the average slope for the CRISP was significantly steeper than for the MPST (*p* = 0.005), indicating that the CRISP was more fatiguing overall.

Notably, 26.1% of participants demonstrated a statistically significant increase in time during the MPST, compared to 41.3% during the CRISP. One child showed improvement over repetitions on the MPST (a 13-year-old boy). The rest of the children slowed down over runs but their individual regression lines were not significantly different from zero.

### 3.8. Association Between Fatigue Metrics

Two sets of correlation analyses between all fatigue outcomes are presented in the Supplementary Materials: Table S1 shows relationships among outcomes derived from the sprint times of the MPST and Table S2 presents those from the CRISP. In the MPST, the slope of individual performance decline showed moderate correlations with both the decrement relative to the ideal sprint performance (DECR; r = −0.529, *p* < 0.001) and the difference between the fastest and slowest sprint (FI; r = −0.504, *p* < 0.001). This indicates that steeper performance declines were generally accompanied by a larger increase in time over the runs. As expected, FI and DECR were strongly associated (r = 0.91, *p* < 0.001), indicating that these two commonly used indicators of fatigue reflect similar performance characteristics in this context. The decay between the first and final sprint showed a strong correlation with the individual slope (r = 0.826, *p* < 0.001), supporting its value as a straightforward measure of performance decline. It was also moderately associated with the R^2^ value of the slope fit (r = 0.578, *p* < 0.001), suggesting that children with a more consistent increase in time tended to show greater total decline. Notably, performance decay was unrelated to initial sprint speed, implying that fatigue during the MPST was not influenced by how fast a participant started but rather by how well they maintained performance across repeated sprints.

Overall, the correlations between the slope of performance decline in the CRISP and the other fatigue indicators were slightly stronger than those observed in the MPST. The slope was moderately to strongly associated with both the decrement relative to the ideal sprint performance (DECR r = −0.71, *p* < 0.001) and the difference between the fastest and slowest sprint (FI r = −0.71, *p* < 0.001), indicating that a steeper decline in speed was consistently linked to greater fatigue across repetitions. FI and DECR, as in the MPST, were strongly correlated (r = 0.927, *p* < 0.001), confirming that these two measures capture closely related aspects of performance deterioration. The decay from the first to the final sprint was strongly associated with the slope of the CRISP (r = −0.897, *p* < 0.001), reinforcing the validity of this simpler metric as a marker of fatigue. Additionally, the decay showed a moderate correlation with the R^2^ value of the slope (r = 0.689, *p* < 0.001), suggesting that participants with a more consistent performance drop also experienced greater overall decline.

## 4. Discussion

The health and fitness of children and adolescents have recently received more attention, prompting the development of various field-based test batteries. Repeated-sprint ability protocols give insight into the capacity to produce and maintain maximal or near-maximal sprint efforts with short recovery intervals, integrating neuromuscular power, anaerobic energy production, and recovery mechanisms. However, the assessment of aspects of anaerobic performance, especially through repeated-sprint ability protocols, has received relatively less emphasis compared to aerobic fitness [30,38]. This study extends earlier work on the CRISP and MPST by analyzing fatigue development over 15 and 30 m distances in children, with a focus on sex differences and the sensitivity of different fatigue indices. Our earlier study was performed in a younger age group [38], limited by a small sample size and could not explore sex-based differences; this study aims to address that gap.

### 4.1. Differences Between the Tests

One key objective was to compare the development of fatigue in the CRISP and the MPST protocols. The CRISP test, which involved a longer sprint time (mean sprint time: 6.39 s vs. 3.37 s), enabled participants to reach higher top speeds, while the MPST was more reflective of initial acceleration capacity. Fatigue, as measured by the decay in speed between first and last sprint (decay) and the linear slope of performance decline (slope IP), was consistently greater in the CRISP. This suggests that longer distances increase fatigue accumulation, likely due to greater reliance on glycolytic involvement. This suggests that CRISP may be more suitable for detecting fatigue in school-based fitness assessments.

Among the fatigue indices, slope IP appeared particularly robust, as it incorporated data from all six sprints, capturing the overall pattern of decline. Decay was highly correlated with both MPST and CRISP slope IP. However, its accuracy depends on the linearity of the performance decay as indicated by the relationship between decay and the R-squared value. Importantly, none of the fatigue indices correlated strongly with initial or mean sprint speed, suggesting that fatigue measurement is independent of sprint performance level.

As shown in prior studies, sprint distance and recovery time affect energy system engagement and fatigue onset [39]. For short efforts (3–6 s), the ATP-CP system predominates, while longer sprints begin to involve glycolysis [40,41]. In repeated-sprint protocols, oxidative contributions rise during recovery intervals, especially when recovery is short [42]. This aligns with the greater fatigue observed in the CRISP, where metabolic demands are expected to be higher due to increased glycolytic involvement. Other studies suggest that during exhaustive exercise lasting 10 s, anaerobic contributions (both ATP-CP and glycolytic) account for 94% of energy supply, while oxidative contributions account for only 6% [40]. However, after repeated-sprint protocols, the contribution from the aerobic system is reported to increase to approximately 40% [39]. These findings suggest that the oxidative system is minimally involved during sprinting and plays a greater role during rest intervals [42].

Interestingly, a subset of participants demonstrated no significant increase in running time based on their slope. This finding could reflect two distinct phenomena. Some children may not have performed at maximal effort, obscuring a large fatigue-related decline. Conversely, others may have had sufficient anaerobic capacity or efficient recovery mechanisms to partly recover during the rest period, leading to non-significant decline, particularly in the shorter MPST where the work–rest ratio was better. As mentioned, our operational definition does not capture the full multidimensional nature of fatigue. Future studies should therefore include measures of perceived exertion, coach ratings, training levels or heart rate responses to help clarify these different performance patterns.

### 4.2. Comparison Between Fatigue Metrics

In this study, four commonly used fatigue indices were compared, which primarily illustrate expected associations among these derived metrics based on the same underlying sprint time data. Consistent with earlier studies, some differences between these metrics emerged. Similar to Tsoukos and Bogdanis [11], the percent sprint decrement was around 50% lower than the Fatigue index. This could likely be because decrement reflects the average decline in performance across the sprints (first and last), whereas the fatigue index focuses on the extremes, that is, between the best and worst run. Lower correlations between some outcomes support earlier conclusions that different indices capture distinct aspects of performance decline [27]. As such, they should not be treated as interchangeable without taking their specific characteristics into account.

Prior studies have reported mixed results regarding sex differences in fatigue indices during repeated-sprint performance, with some showing a lower fatigue index in females [43,44], while others found no sex differences [11,45]. The decay and slope IP were more sensitive in detecting fatigue differences between protocols and sexes, suggesting they may be better suited for field applications in youth settings.

### 4.3. Comparison Between Males and Females

Sex-based differences were evident in this study. Females ran more slowly in both tests and showed greater fatigue in the CRISP test than males. These findings are consistent with studies showing that males outperform females in sprinting and anaerobic tasks before and after puberty [11,46]. Differences in muscle mass and power output are likely contributors. Several authors have stated that increased anaerobic power is mainly related to the development of (lean) muscle mass [47,48]. Although the natural physiological adaptations in the structure of men and women are similar in many respects, there are anatomical and physiological differences [49]. Skeletal muscle constitutes approximately 50% of body mass in men and 40% in women [50]. The differences in muscle mass and leg length likely contribute to variations in running performance and fatigue resistance, with males generally showing higher sprint performance and greater endurance [51]. Additionally, it has been reported that males exhibited better ATP and glycolytic capacities due to higher lactate dehydrogenase levels and a better creatine phosphate system, which might result in higher anaerobic performance [52]. However, this gap between the sexes may not be present in younger children. Temfemo et al. [10] found no sex differences in repeated sprinting in 6- to 8-year-olds, and studies by Taylor et al. [53] and Falk and Dotan [54] showed that children recover more quickly than adolescents or adults, likely due to a more aerobic energy profile. These findings underscore the importance of age and developmental stage when evaluating RSA and fatigue. In addition to these biological factors, sociocultural and motivational factors such as prior training exposure, encouragement, or effort regulation may differ by sex at this age, especially in a school-based setting.

### 4.4. Limitations of the Study and Suggestions for Future Research

This study has several limitations. The sample recruitment may limit the generalizability of the findings, and the training status, which likely affects fatigue responses, was not controlled. An index of biological maturation should have been included, particularly when examining sex differences in this age group, given the substantial and variable developmental changes occurring during this period. Without maturation indicators, sex-based fatigue characteristics observed in this study may be partly attributable to differences in developmental stage rather than sex alone.

Another limitation of this study is that it only reflects performance fatigability, while perceived or central fatigue were not measured. Only one task variable (running distance) was manipulated, which limits generalizability because fatigue is known to be multifaceted and highly task-dependent [55].

Lastly, although manual timing is commonly used in most fitness tests administered in school- and field-based pediatric research, small timing inaccuracies may influence fatigue indices and reduce their sensitivity to detect subtle performance changes due to measurement error. To enhance precision, the use of electronic timing systems is advised.

Future research should investigate how factors such as biological maturation, training status or experience, and task constraints affect fatigue patterns in youth.

### 4.5. Practical Implication

The findings of this study offer practical guidance for educators and coaches working with children aged 9–14 years. First, the greater fatigue observed during 30 m sprints compared to 15 m sprints suggests that longer sprint distances may be more effective for assessing anaerobic endurance and fatigue in youth. This is particularly relevant for designing fitness assessments or training drills that aim to challenge and develop repeated-sprint ability (RSA). It should be taken into consideration that RSA fatigue is fundamentally designed to assess anaerobic glycolytic stress, which may not be fully developed in younger populations. Therefore, RSA fatigue in children may reflect developmental aspects rather than stable or trainable anaerobic characteristics.

Second, the observed sex-based differences highlight the importance of tailoring sprint-based activities to account for developmental and physiological differences. PE teachers should consider adjusting sprint distances, recovery intervals, or repetition numbers to ensure equitable challenge and engagement across sexes.

Third, the study supports the use of a simple fatigue metric such as the decay (first vs. last sprint) as a valid and practical tool for monitoring fatigue during RSA in school settings. This metric requires minimal equipment and can be easily implemented during regular PE classes, making it accessible for educators.

Finally, incorporating repeated-sprint activities into PE curricula aligns with children’s natural play patterns. Educators and coaches are encouraged to integrate structured sprint protocols to enhance both fitness and engagement, while using fatigue metrics to monitor progress and adapt instruction.

These recommendations should be interpreted as indicative and adapted according to local educational, cultural, and regional conditions.

## 5. Conclusions

By comparing RSA performance across two sprint protocols, this study highlights the interaction between sprint distance, fatigue measurement and sex in children aged 9–14. Fatigue was more pronounced in the longer CRISP test, especially in females, and was most clearly detected using the decay and slope IP methods. These findings suggest that the choice of fatigue metric significantly influences conclusions about sex differences and fatigue in youth sprinting. Slope, the only metric which accounts for individual performance across all runs, suggests it to be a better fatigue metric for group comparison. However, slope correlated strongly with performance decay. Therefore, comparing running time between the first and last run in repeated sprinting seems a valid yet straightforward way to measure fatigue when testing repeated-sprinting ability in youth.

## Figures and Tables

**Table 1:** Age distribution by sex.

	Males		Female	
AGE	Frequency	Percent	Frequency	Percent
9	10	14.3	11	15.7
10	11	15.7	19	27.1
11	14	20	11	15.7
12	14	20	11	15.7
13	13	18.6	15	21.4
14	8	11.4	3	4.3
TOTAL	70	100	70	100

**Table 2:** Means, medians, SDs (standard deviations), and IQRs (interquartile ranges) of all repeated-sprint outcomes for males and females.

	MPST Time (s)	CRISP Time (s)	MPST Speed (m/s)	CRISP Speed (m/s)	Decay MPST (%)	Decay CRISP (%)	FI MPST (%)	FI CRISP (%)	Decr MPST (%)	Decr CRISP (%)	Slope MPST (beta)	Slope CRISP (beta)
Males (n)	70	70	70	70	70	69	70	70	70	70	69	69
Missing	0	0	0	0	0	1	0	0	0	0	1	1
Mean	3.27	6.22	4.64	4.86	4.35	5.94	13.22	12.03	6.24	5.69	−0.045	−0.050
Median	3.31	6.24	4.54	4.81	3.89	4.35	11.40	10.10	5.45	4.86	−0.048	−0.053
SD	0.30	0.42	0.45	0.33	7.29	6.84	6.17	6.88	3.43	3.59	0.055	0.056
IQR Low	3.04	5.92	4.33	4.63	−0.69	1.80	8.65	6.87	3.67	3.20	−0.009	−0.008
IQR High	3.47	6.48	4.94	5.08	8.01	8.97	16.22	15.17	7.81	7.51	−0.081	−0.085
Females (n)	70	70	70	70	70	70	70	70	69	70	69	69
Missing	0	0	0	0	0	0	0	0	1	0	1	1
Mean	3.48	6.57	4.36	4.62	7.01	11.01	14.99	16.38	6.87	8.14	−0.057	−0.093
Median	3.52	6.60	4.28	4.55	7.18	9.36	12.29	14.70	6.40	6.68	−0.059	−0.090
SD	0.34	0.63	0.45	0.46	10.07	9.71	7.88	10.35	3.50	6.08	0.057	0.065
IQR Low	3.22	6.21	4.04	4.33	1.15	4.81	9.25	9.61	4.34	4.38	−0.020	−0.046
IQR High	3.72	6.96	4.67	4.86	11.16	16.74	19.02	19.89	8.50	9.84	−0.084	−0.135

**Table 3:** Test statistics for the Wilcoxon signed-rank test for the comparison between outcomes of the MPST and CRISP; test statistics for the Mann–Whitney U test for the comparison between males and females on the MPST and the CRISP.

Variable	Test Differences MPST and CRISP (z- and *p*-Values)	Sex Differences MPST (z-, *p*-Values and Effect Size)	Sex Differences CRISP (z-, *p*-Values and Effect Size)
Running time	z = 10.27, *p* < 0.001	z = 3.77, *p* < 0.001, d 0.672	z = 4.00, *p* < 0.001, d 0.717
Running speed	z = −7.67, *p* < 0.001	z = −3.77, *p* < 0.001, d 0.672	z = −3.95, *p* < 0.001, d 0.708
Decay start finish	z = −2.56, *p* = 0.010	z = 1.88, *p* = 0.06, d 0.322	z = 3.69, *p* < 0.001, d 0.599
Fatigue index	z = −0.86, *p* = 0.39	z = 1.31, *p* = 0.19, d 0.222	z = 2.87, *p* = 0.004, d 0.500
Sprint decrement	z = −0.49, *p* = 0.63	z = 1.44, *p* = 0.15, d 0.195	z = 2.89, *p* = 0.004, d 0.504
Beta slope IP	z = 2.83, *p* = 0.005	z = −1.08, *p* = 0.28, d 0.130	z = −3.92, *p* < 0.001, d 0.774

## Data Availability

All data needed for the analysis are available within the article. Datasets used and/or analyzed during the current study are available from the corresponding author on reasonable request.

## Supplementary Materials

The following supporting information can be downloaded at https://www.mdpi.com/article/10.3390/sports14030104/s1, Table S1: Spearman’s rho Correlations for outcomes of the MPST; Table S2: Spearman’s rho Correlations for outcomes of the CRISP.
