# Supplementary material for: Sex-Based Differences in Fatigue During Repeated Sprinting in 9- to 14-Year-Old Children Are Task- and Metric-Dependent
Source: Sports (Basel). 2026 Mar 5;14(3):104. doi: 10.3390/sports14030104 (PMC13029879; doi:10.3390/sports14030104)
Supplement: Supplementary file 1 [file sports-14-00104-s001.zip › sports-4111291-supplementary.pdf]

Table S1. Spearman's rho Correlations for outcomes of the MPST.

Variables	Time (s)	Decay	FI	DECR	Slope IP	R-Square Fit
**Decay**	.229**		.327**	.378**	- .826**	.578**
**FI**	.065	.327**		.901**	- .504**	.019
**DECR**	.000	.378**	.901**		- .529**	.096
**Slope IP**	-.037	- .826**	- .504**	- .529**		- .672**
**R Square Fit**	.008	.578**	.019	.096	- .672**	
**Constant Y**	-.892**	.132	.119	.180*	- .348**	.237**

** Correlation is significant at the 0.01 level * Correlation is significant at the 0.05 level. Decay; Decay Start to Finish; FIS: Fatigue Index; DECR: sprint decrement; Slope IP: Slope of the individual performance decrement.

Table S2. Spearman's rho Correlations for outcomes of the CRISP.

Variables	Time (s)	Decay	FI	DECR	Slope IP	R-Square Fit
**Decay**	.299**		.671**	.658**	-.897**	.689**
**FIS**	.323**	.671**		.927**	-.710**	.289**
**DECR**	.294**	.658**	.927**		-.710**	.317**
**Slope IP**	-.185*	-.897**	-.710**	-.710**		-.758**
**R Square Fit**	.034	.689**	.289**	.317**	-.758**	
**Constant Y**	-.822**	.196*	.073	.100	-.349**	.342**

** Correlation is significant at the 0.01 level. * Correlation is significant at the 0.05 level. Decay; Decay Start to Finish; FIS: Fatigue Index; DECR: sprint decrement; Slope IP: Slope of the individual performance decrement.
